# Study of Dietary Emodin on Immune Defense in *Megalobrama amblycephala* against *Aeromonas hydrophila*

**DOI:** 10.3390/vetsci10090588

**Published:** 2023-09-21

**Authors:** Yuan-Yuan Zhang, Peng Xu, Xiao-Li Wang, Li-Ping Song, Jun Wu, Bing-Li Wang, Bin Hu, Shu-Quan Mao, Bo Liu, Xian-Ping Ge

**Affiliations:** 1 Shandong Key Laboratory of Freshwater Aquatic Genetics and Breeding, Shandong Freshwater Fisheries Research Institute, No. 162 Jiefang Road, Jinan 250013, China; yyuanzhang2008@163.com (Y.-Y.Z.); wangxiaoli@163.com (X.-L.W.); wujun@163.com (J.W.);; 2 Key Laboratory of Freshwater Fisheries and Germplasm Resources Utilization, Freshwater Fisheries Research Center, Chinese Academy of Fishery Sciences, Ministry of Agriculture, No. 9 Shanshui East Road, Wuxi 214081, China

**Keywords:** *Aeromonas hydrophila*, *Megalobrama amblycephala*, emodin, nonspecific immunity, antibacterial

## Abstract

**Simple Summary:**

This study focuses on the Wuchang bream (*Megalobrama amblycephala*) as the research subject, aiming to investigate the effects of emodin on the total bacterial count of *Aeromonas hydrophila* and the immune response in various tissues of Wuchang bream following infection. The experimental diets were made by supplementing emodin at 0, 30, 100, and 150 mg kg^−1^ to a basal (control) diet, respectively, and fed to fish with an initial weight of 50.4 ± 2.35 g. All fish were divided into five experimental groups: uninfected fish fed with basal control diet (negative control, NC), infected fish fed with the diet supplemented with 0 (positive control group, PC), 30 (30), 100 (100), and 150 mg/kg (150) of emodin. Except for the negative control group, all other groups were injected with *A. hydrophila* at a concentration of 1 × 10^6^ CFU/mL. The experiment was conducted over a period of 14 days, with sampling at various time points. The results of the experiment demonstrated that the total bacterial count of *Aeromonas* in the kidney, blood, and liver tissues of infected Wuchang bream was significantly affected by the dosage of added emodin and the feeding duration. Additionally, the immune response of Wuchang bream following intraperitoneal infection with *A. hydrophila* was also significantly influenced by emodin (*p* < 0.05). In conclusion, the addition of 100 mg/kg of emodin to the diet could enhance the Wuchang bream’s resistance to *A. hydrophila* infection by reducing the total bacterial count of pathogenic bacteria in tissues, increasing the activity of relevant immune enzymes and promoting the secretion of cytokines. This study provides a theoretical basis for practical production.

**Abstract:**

This experiment aimed to investigate the effects of emodin on the total bacterial count and immune response in various tissues of Wuchang bream infected with *A. hydrophila*. The experimental diets were made by supplementing emodin at 0, 30, 100, and 150 mg kg^−1^ to basal (control) diet, respectively, and fed to fish with an initial weight of 50.4 ± 2.35 g. All fish were divided into five experimental groups: uninfected fish fed with basal control diet (negative control, NC), infected fish fed with the diet supplemented with 0 (positive control group, PC), 30 (30), 100 (100), and 150 mg/kg (150) of emodin. The fish were reared for 14 days and sampled at different time points. The results showed that the total bacterial count in the kidney, blood, and liver tissues of Wuchang bream infected with *A. hydrophila* was significantly affected by the supplementation and feeding time of emodin. At the beginning of the experiment, the difference in total bacterial count among the groups was not significant. On day 1, the total bacterial count in all groups was significantly higher (*p* < 0.05) than that in the negative control group. On day 4, the total bacterial count in all the emodin groups was significantly reduced, and the best bactericidal effect was observed in the 100 mg kg^−1^ group. In addition, emodin had a significant effect on the immune response of Wuchang bream after infection with *A. hydrophila* (*p* < 0.05). Compared with the other groups, the respiratory burst activity, tumor necrosis factor-α (TNF-α), interleukin-1 (IL-1) content, and white blood cell count (WBC) in the 100 and 150 mg kg^−1^ groups could be restored to normal levels in the shortest time (*p* < 0.05). Furthermore, this study also measured the complement alternative pathway activity (ACH_50_), plasma superoxide dismutase (SOD) activity, and malondialdehyde (MDA) content of the fish. The results showed that supplying 100 mg kg^−1^ emodin to the diet could significantly (*p* < 0.05) increase the ACH_50_ activity of the fish. Compared with the positive control (PC) group, the addition of emodin to the diet can inhibit the decrease in SOD activity and the increase in MDA content in the plasma of infected Wuchang bream. In conclusion, supplying 100 mg kg^−1^ emodin to the diet can enhance the ability of Wuchang bream to resist *A. hydrophila* infection by reducing the total bacterial count in tissues, increasing the activity of related immune enzymes, and promoting the secretion of cytokines. This provides a theoretical basis for production practice.

## 1. Introduction

*Aeromonas hydrophila*, a water-loving bacterium, is widely distributed in nature and commonly found in freshwater, sewage, sludge, soil, and human feces [1]. It has a wide range of pathogenicity and can infect various animals, including fish, amphibians, reptiles, birds, and mammals, causing septicemia or local infections, such as skin ulcers. In addition, during hot summers, it can cause large-scale outbreaks of diseases in aquatic animals, especially fish [2]. Once infected, fish can suffer from high mortality rates, causing unavoidable losses for aquaculturists [3]. To prevent such occurrences, a large number of chemical drugs and antibiotics have been widely used in aquaculture. However, the misuse of antibiotics can lead to drug resistance and drug residues in organisms, which not only have a negative impact on the treatment of fish diseases but also pose significant harm to human health. Chinese herbal medicine, as a new type of natural medicine, has attracted much attention due to its natural, residue-free, low toxicity, non-resistance, and low-cost advantages [4,5]. Therefore, exploring the best preventive medicine additives to enhance the resistance of fish to *A. hydrophila* infection has become an urgent issue.

Emodin is the main effective monomer extracted from the roots and rhizomes of *Polygonaceae* plants, such as *Reynoutria japonica Houtt* and *Rheum officinale Baill*. Many beneficial functions have been reported for emodin, such as antibacterial and anti-inflammatory [6], antioxidation and scavenging free radicals [7], reducing blood lipid [8], protecting the liver [9], and regulating immunity [10]. Wuchang bream (*Megalobrama amblycephala*) is a major species of freshwater fish cultured in China. There were studies indicated that anthraquinone extract (a mixture of emodin, chrysophanol, and rhein) [11] and emodin [12] could promote the growth and enhance the non-specific immunity and high-temperature tolerance of Wuchang bream (*Megalobrama amblycephala*). We previously reported that emodin has good in vitro antibacterial ability against *A. hydrophila* [13]. Additionally, as an immunostimulant, emodin can, to some extent, improve the growth performance of Wuchang bream, enhance non-specific immunity, and exert antioxidant effects [14]. However, its effect as a preventive drug additive on the resistance of Wuchang bream to *A. hydrophila* infection needs further research. 

Given this, this experiment explores the effects of different dosages of emodin on the resistance of *M. amblycephala* to infection by *A. hydrophila* based on preliminary experiments, clarifies the relationship between the dosage of emodin and the anti-infection ability of *M. amblycephala*, reveals the changing patterns of the total bacterial count of *Aeromonas* and various immune indicators in various tissues of *M. amblycephala* with the dosage of emodin and the sampling time, elucidates the mechanism of emodin in *M. amblycephala*, and provides theoretical basis and technical support for the use of therapeutic drug additive in production practice.

## 2. Materials and Methods

### 2.1. Experimental Diets

The formulation and proximate composition of the basal diet are shown in Table 1. The basal diet was supplemented with 0 (control), 30, 100, and 150 mg emodin kg^−1^ diet, respectively. The emodin used had a purity of over 99% and was provided by Feida Chemical Reagent Company (Xian, China). To prepare the experimental diets, the ingredients were ground into a fine powder using a 60-mesh sieve. Dry ingredients were mixed together, and then oil and water were added (40% *v*/*w*) to form a soft dough. The dough was then pelleted using a laboratory pellet machine and dried in a forced-air oven at room temperature. After drying, the diets were broken into smaller pieces and sieved into the proper pellet size. All diets were stored at −20 °C until used.

### 2.2. Experimental Fish and Breeding Management

Wuchang bream were obtained from the Nanquan fish farm of the Freshwater Fisheries Research Center, Chinese Academy of Fishery Sciences. Prior to the experiments, fish were fed with a basal diet for an acclimation period of 15 days. Fish were hand-fed with the test diets three times (08:00, 12:00, and 16:00) per day until apparent satiation for 14 days. During the experiment period, the juveniles were retained under a natural photoperiod. The water temperature was controlled at 26 ± 1 °C over the experimental period. pH fluctuated from 7.2 to 7.8, and ammonia nitrogen was lower than 0.05 mg L^−1^. Dissolved oxygen concentration was more than 6 mg L^−1^.

### 2.3. Challenge Test

#### 2.3.1. Preparation of Fungal Broth

Gram-negative *A. hydrophila* was originally isolated from the infected fingerling *M. amblycephala*. The seven-day LC_50_ was determined by intraperitoneal injection of 48 fish with graded concentrations of *A. hydrophila* (10^6^, 10^7^, 10^8^, 10^9^ and 10^10^ CFU mL^−1^) at 24 °C, yielding a day 7 LC_50_ of 5 × 10^6^ CFU mL^−1^. Based on the experimental results, it was determined that the bacterial concentration of 1 × 10^6^ CFU mL^−1^ causes disease but does not result in mortality in the Wuchang bream.

Under aseptic conditions, a strain of aquatic *A. hydrophila* stored at −80 °C was inoculated onto LB solid agar plates and cultured for 20 h at 28 °C in a constant temperature incubator. A single colony was then selected and inoculated into 5 mL of LB liquid medium and cultured overnight at 28 °C on a constant temperature shaker at 180 rpm min^−1^. The bacterial suspension was diluted using a sterile LB liquid medium in a 10-fold series, and a bacterial suspension of 1 × 10^6^ CFU mL^−1^ was selected for the virulence test.

#### 2.3.2. Experiment Design

All fish (average weight, 50.4 ± 2.35 g) were divided into five experimental groups of 25 each in triplicate into Group I: uninfected fish fed with a basal control diet (negative control, NC), Group II: infected fish fed with a basal control diet (positive control, PC), Group III: infected fish fed with the diet containing 30 mg emodin kg^−1^ (30), Group IV: infected fish fed with the diet containing 100 mg emodin kg^−1^ (100), and Group V: infected fish fed with the diet containing 150 mg emodin kg^−1^ (150). 

### 2.4. Sample Collection

On days 0, 1, 4, 7, and 14 of the experiment, three fish were randomly selected from each tank and anesthetized with MS-222 (100 mg L^−1^, tricaine methanesulfonate, Sigma, Shanghai, China) diluted with pH buffer. Under aseptic conditions, blood was collected by the caudal venipuncture using 1 mL heparinized syringes. Finally, the liver and kidneys were removed from three fish. A small piece of the liver was put into Bouin’s solution for histological examination (On days 1 and 7 of the experiment). After collection, a portion of the blood samples were used for bacterial counting along with the isolated liver and kidney, while another portion of the blood samples were used for the measurement of relevant immunological indicators. Among them, 50 µL whole blood was used for the analysis of respiratory burst activity. A small portion of whole blood was used for the analysis of white blood cell count. The remaining whole blood was centrifuged (3500× *g*, 4 °C, 10 min) to obtain plasma (stored at −80 °C) for the analysis of SOD activity, MDA, TNF, IL-1 contents, and complement activity.

### 2.5. Colony Enumeration

Under aseptic conditions, blood was extracted from Wuchang bream, and some kidney and liver tissues were dissected, separated, and weighed for later use. The tissues were homogenized using a sterilized homogenizer and made into a bacterial suspension by adding 1 mL of sterilized physiological saline and shaking evenly. Blood, liver, and kidney bacterial suspensions were used as the original samples and were diluted into 4 gradients of 10^−1^, 10^−2^, 10^−3^, and 10^−4^ by the 10-fold dilution method. Then, 0.1 mL of each sample, including the original and each dilution gradient, was spread on an RS selective culture medium (Table 2 for preparation method) and incubated overnight in a constant temperature incubator for 20 h, followed by counting. The sampling time was used as the horizontal coordinate, and the logarithm of the number of bacteria counted was used as the vertical coordinate to make a graph.

### 2.6. Immunological Assays

#### 2.6.1. White Blood Cell Count (WBC)

White blood cell count (WBC) of obtained blood samples was directly measured using an Auto Hematology Analyzer (BC-5300Vet, Mindray, Shenzhen, China) with a test kit from Shenzhen Mindray Medical International Co., Ltd. (Shenzhen, China).

#### 2.6.2. Respiratory Burst Activity

Respiratory burst activity of phagocytes was detected with nitroblue tetrazolium (NBT, Shanghai Reagent Corp., Shanghai, China) following the methods described by Secombes et al. [15] and Ai et al. [16]. Absorbance at 630 nm was measured with a Model Multiskan spectrum (Thermo, Shanghai, China) using KOH/DMSO as a blank. Respiratory burst was expressed in terms of NBT reduction in 100 mL of cell suspension. 

#### 2.6.3. Plasma Superoxide Dismutase (SOD) and Malondialdehyde (MDA) Assay

SOD activity and MDA content were measured using xanthine oxide [17] and barbituric acid reaction chronometry [18], respectively. These kits were purchased from the Nanjing Jiancheng Bioengineering Institute of China.

#### 2.6.4. Plasma Tumor Necrosis Factor-α (TNF-α) and Interleukin-1 (IL-1) Assay

According to the reference of Zhao et al. [19] and Li et al. [20], the hemolymph modules involving tumor necrosis factor-α (TNF-α) and interleukin-1 (IL-1) were estimated utilizing an enzyme-linked immunosorbent assay (ELISA) kit (Shanghai Enzyme-linked Biotechnology Co., Ltd., Shanghai, China) following the manufacturer’s protocols whereby from each group, nine blood samples were checked.

#### 2.6.5. Complement Activity Assay

The complement activity was measured by using sheep red blood cells (SRBC, Biomedics, Shanghai, China) as targets [21]. The degree of 50% hemolysis (ACH_50_) units mL^−1^ and the lysis curve of each sample were obtained separately.

### 2.7. Histological Examination

The liver was excised from different fish groups and processed as described by Khan and Parvez [22]. A physiological saline solution (0.75% NaCl) was used to rinse and clean the tissues. They were fixed in aqueous Bouin’s solution (75 mL saturated picric acid, 25 mL formaldehyde (37–40%), and 5 mL glacial acetic acid) for 24 h. Subsequently, liver tissue was processed through a graded series of alcohols, cleared in xylene, and embedded in paraffin wax. Sections were cut at 4–6 µm thickness using an 820-Spencer rotatory microtome, stained with hematoxylin-eosin (dissolved in 70% alcohol) [23], and were mounted in Canada balsam. Thin sections (20 μm) were stained with hematoxylin-eosin and observed with an Olympus BX61 photo-microscope (Beijing Hede Technology Corp., Beijing, China) (×1000). 

### 2.8. Statistical Analysis

All data are presented as means ± S.E. (standard error of the mean). Data were logarithmically transformed before subjection to one-way analysis of variance (ANOVA) using SPSS 17.0. When the overall treatment effect was found to be significantly different, the LSD multiple range tests were conducted to compare the means between the levels of emodin and time treatment, respectively. The level of significant difference was set at *p* < 0.05.

## 3. Results

### 3.1. Enumeration of Monobacterial Colonies of the Genus Aeromonas spp.

**Figure 1:**
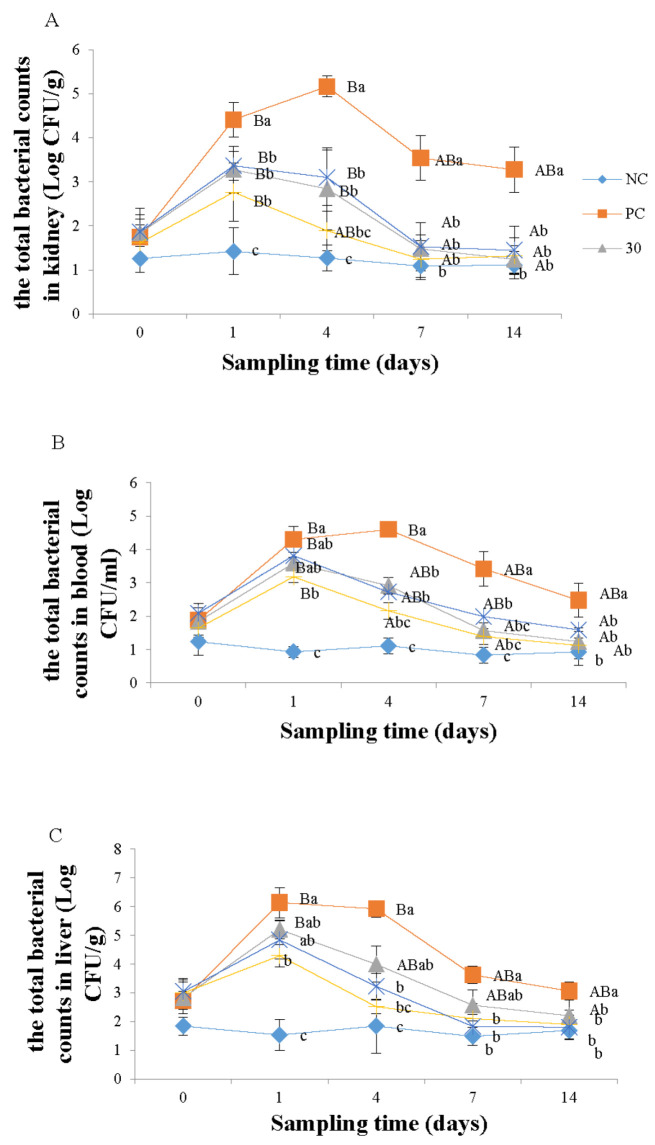
Effects of emodin on total *Aeromonas* counts in *Megalobrama amblycephala* kidney (**A**), blood (**B**) and liver (**C**) after intraperitoneal injection. Note: Data are expressed as means ± SEM (*n* = 9). Diverse little letters show significant differences (*p* < 0.05) in different dosage groups of each sampling point, and diverse capital letters show significant differences (*p* < 0.05) at different times in Duncan’s multiple range test.

Figure 1A shows the effect of emodin on the total number of *Aeromonas* in the kidneys of Wuchang bream infected with *A. hydrophila*. At the beginning of the experiment, there was no significant difference in the total number of bacteria among the groups (*p* > 0.05). On day 1 of the experiment, the total number of bacteria in all groups increased significantly compared to the negative control group (*p* < 0.05), and the positive control group was significantly higher (*p* < 0.05) than the 30, 100, and 150 mg kg^−1^ emodin groups. On day 4, there was no significant difference (*p* > 0.05) in the total number of bacteria between the 100 mg kg^−1^ emodin group and the negative control group, but the total number of bacteria in the 30 and 150 mg kg^−1^ emodin groups was significantly higher (*p* < 0.05) than the negative control group and significantly lower than the positive control group. On day 7, the total number of bacteria in the 30, 100, and 150 mg kg^−1^ emodin groups was significantly lower (*p* < 0.05) than the positive control group, with no significant difference between these groups and the negative control group. After 14 days of feeding, there was no significant difference among the groups (*p* > 0.05). In addition, the total bacterial count of *A. hydrophila* in the kidneys of infected Wuchang bream was significantly influenced by the feeding time. Both the PC group and emodin groups exhibited a trend of initially increasing and then decreasing bacterial counts. Specifically, the bacterial counts in the PC group, the 30, and the 150 mg/kg groups were significantly higher (*p* < 0.05) than at the beginning of the experiment on days 1 and 4, and they started to decrease significantly from day 7 onwards. The bacterial counts in the 100 mg/kg group were significantly higher (*p* < 0.05) on day 1 of the experiment compared to the initial count, but there was no significant difference (*p* > 0.05) in bacterial count on day 4 compared to the initial count.

As shown in Figure 1B, the total number of *Aeromonas* in the blood of infected Wuchang bream was significantly affected by the feeding time. In the positive control group, the total number of bacteria in the blood of fish increased greatly, reaching the level of 10^4^ on the first day of the experiment and reaching 10^5^ on the fourth day, followed by a downward trend. The total number of blood bacteria in the 30, 100, and 150 mg kg^−1^ groups all showed an initial increase followed by a decrease, reaching the highest level on the first day of the experiment, followed by a downward trend. Additionally, the 100 mg kg^−1^ group showed a significant decrease (*p* < 0.05) in blood bacteria compared to the other groups on the fourth day. Moreover, the total number of *Aeromonas* in the blood of infected Wuchang bream was also significantly affected by the supplementation of emodin. On day 1 of the experiment, the total number of *Aeromonas* in the negative control group was significantly lower than (*p* < 0.05) the other four groups and the 100 mg kg^−1^ emodin group was significantly lower than (*p* < 0.05) the positive control group. On day 4, the total number of *Aeromonas* in the positive control group was significantly higher than (*p* < 0.05) the other four groups, and compared to the negative control group, the total number of *Aeromonas* in the 30 and 150 mg kg^−1^ groups significantly increased, while there was no significant difference (*p* > 0.05) in the 100 mg kg^−1^ group. On the 7 and 14 days, compared to the positive control group, the total number of *Aeromonas* in the other four groups significantly decreased (*p* < 0.05).

As shown in Figure 1C, the total number of *Aeromonas* in the liver of infected Wuchang bream was significantly affected by the feeding time of emodin. The negative control group had relatively stable and low *Aeromonas* counts, while the other four groups showed an initial increase followed by a decreasing trend. Among them, the bacterial count in the positive control group was significantly higher than at the beginning of the experiment on days 1 and 4 (*p* < 0.05), but there was no significant difference compared to the initial count from day 7 onwards (*p* > 0.05). The bacterial count in the 30 mg kg^−1^ group significantly increased on day 1 of the experiment (*p* < 0.05), but there was no significant difference compared to the initial count from day 4 onwards (*p* > 0.05). The bacterial count in the 100 and 150 mg kg^−1^ groups showed no significant differences at all time points (*p* > 0.05). In addition, the total bacterial count of *Aeromonas* in the liver of infected Wuchang bream was influenced by the dosage of emodin. At the beginning of the experiment, the total *Aeromonas* count in the negative control group was lower but not significantly different (*p* > 0.05) from the other four groups. On day 1, the total *Aeromonas* count in the other four groups increased significantly (*p* < 0.05) compared to the negative control group, and the 100 mg kg^−1^ group was significantly lower (*p* < 0.05) than the positive control group, while there was no significant difference among the other groups (*p* > 0.05). On day 4, the total *Aeromonas* count in the 100 and 150 mg kg^−1^ groups decreased significantly (*p* < 0.05) compared to the positive control group, and the total *Aeromonas* count in the 30 mg kg^−1^ group was lower but not significantly different (*p* > 0.05) from the positive control group. On day 7, the total *Aeromonas* count in the 100 and 150 mg kg^−1^ groups remained significantly lower (*p* < 0.05) than the positive control group. After the 7th day, the total *Aeromonas* count in all groups tended to stabilize, and by the 14th day, there was no significant difference among the groups (*p* > 0.05).

### 3.2. Immune Response

**Figure 2:**
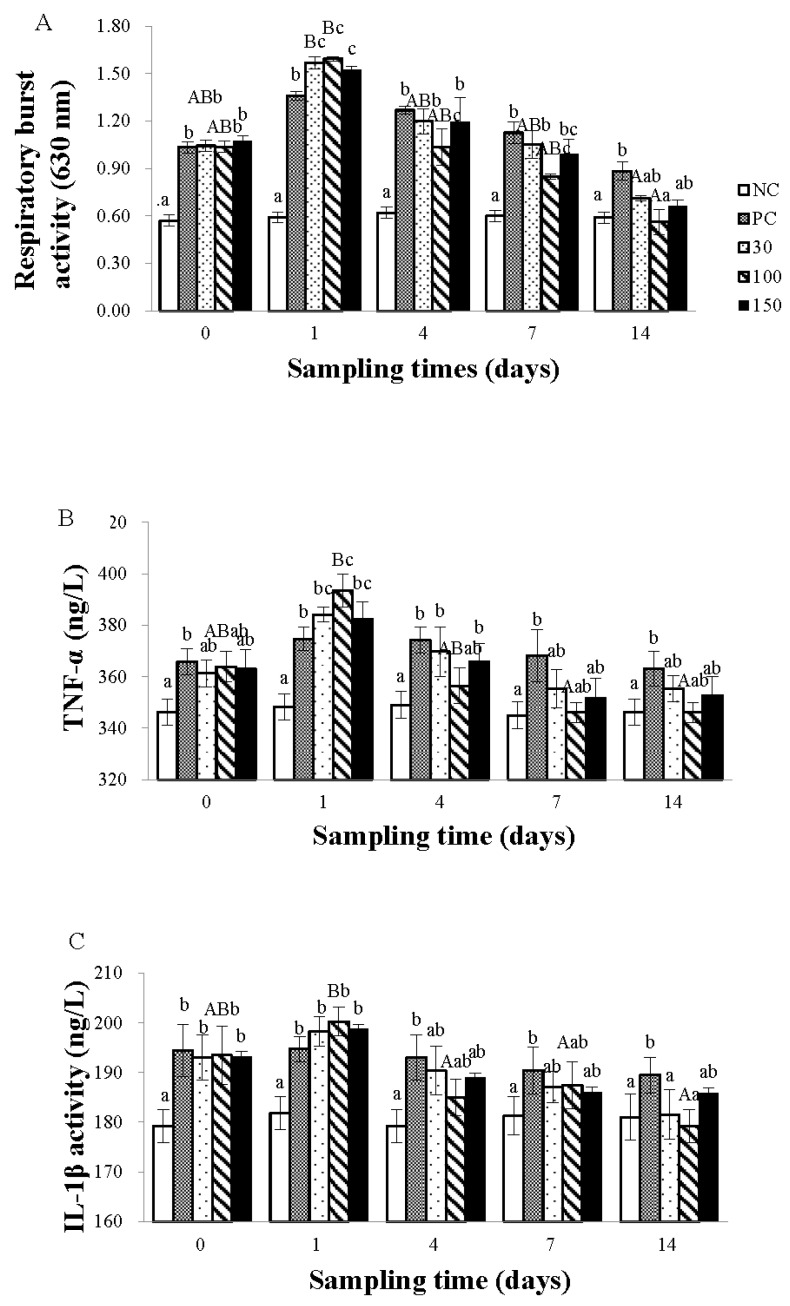
Effects of emodin on Respiratory burst activity (**A**), TNF-α (**B**), and IL-1β (**C**) contents in *Megalobrama amblycephala* after intraperitoneal injection. Note: Data are expressed as means ± SEM (*n* = 9). Diverse lowercase letters show significant differences (*p* < 0.05) in different dosage groups of each sampling point, and diverse capital letters show significant differences (*p* < 0.05) at different times in Duncan’s multiple range test.

As shown in Figure 2A, the respiratory burst activity of the positive control group and the emodin groups were both influenced by the feeding time of emodin, showing a trend of initial increase followed by a decrease. Among them, the effect of feeding time was most significant in the 30 and 100 mg kg^−1^ emodin groups (*p* < 0.05). The respiratory burst activity on the 14th day of the experiment was significantly lower *(p* < 0.05) than on the 1st day, with no significant differences observed at other time points (*p* > 0.05). In addition, the respiratory burst activity of infected Wuchang bream was influenced by the dosage of emodin. At the beginning of the experiment, the respiratory burst activity of the infected groups was significantly higher than that of the negative control group (*p* < 0.05); on day 1, the respiratory burst activity of the 30, 100, and 150 mg kg^−1^ groups was significantly higher than that of the positive control group (*p* < 0.05); on day 4, the respiratory burst activity of the infected groups decreased, and the respiratory burst activity of the negative control group was still significantly lower than that of the other four groups (*p* < 0.05). In addition, the respiratory burst activity of the 100 mg kg^−1^ group was significantly lower than that of the positive control group and the 30 and 150 mg kg^−1^ groups (*p* < 0.05). On day 7, the respiratory burst activity of the 100 mg kg^−1^ group was significantly (*p* < 0.05) lower than that of the positive control group and the 30 mg kg^−1^ group, and there was no significant difference with the 150 mg kg^−1^ group; on day 14, the respiratory burst activity of the 100 mg kg^−1^ group was significantly lower (*p* < 0.05) than that of the positive control group, and there was no significant difference among the other groups.

As shown in Figure 2B, the TNF-α content in the plasma of infected Wuchang bream was significantly influenced by the feeding time of emodin, exhibiting a trend of first increasing and then decreasing. Among them, the 100 mg kg^−1^ group showed the most significant change (*p* < 0.05). The TNF-α content on the 7th and 14th days of the experiment was significantly lower (*p* < 0.05) than on the 1st day, with no significant differences observed at other time points (*p* > 0.05). In addition, the TNF-α content in the plasma of infected Wuchang bream was influenced by the dosage of emodin. At the beginning of the experiment, the TNF-α content of the negative control group was significantly lower than (*p* < 0.05) that of the positive control group, and there was no significant difference (*p* > 0.05) compared to the emodin groups. On day 1, the TNF-α content of the infected groups was significantly higher than that of the negative control group (*p* < 0.05), and the 100 mg kg^−1^ group was significantly higher than the positive control group (*p* < 0.05), while there was no significant difference between the 30 and 150 mg kg^−1^ groups and the positive control group (*p* > 0.05). On day 4, the TNF-α content of the emodin groups was significantly reduced compared to the 1st day (*p* < 0.05). The TNF-α content of the 30, 150 mg kg^−1^ groups and the positive control group were still significantly higher than (*p* < 0.05) that of the negative control group, while there was no significant difference between the 100 mg kg^−1^ group and the negative control group. On days 7 and 14, there was no significant difference in TNF-α content between the emodin group and the negative control group (*p* > 0.05).

As shown in Figure 2C, the trend of changes in plasma IL-1β levels with feeding time is basically the same as that of TNF-α levels. At the beginning of the experiment, the IL-1β levels in the negative control group were significantly lower than those in the other four groups (*p* < 0.05). On day 1, the IL-1β levels in all groups except the negative control group were significantly increased compared to the beginning of the experiment (*p* < 0.05). Compared with the first day, the IL-1β levels in the 100 mg kg^−1^ emodin group were significantly reduced on the fourth day (*p* < 0.05), while the IL-1β levels in the positive control group remained higher than those in the negative control group, and there was no significant difference (*p* > 0.05) among the other groups. On day 7, the IL-1β levels in the positive control group were still significantly higher than those in the negative control group, and there was no significant difference (*p* > 0.05) among the other groups. After the seventh day, the changes in IL-1β levels in each group tended to stabilize.

**Figure 3:**
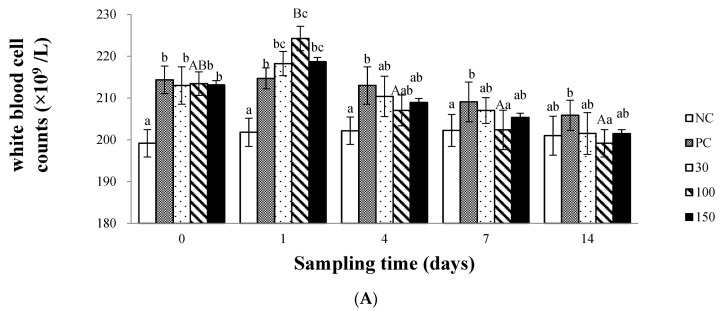

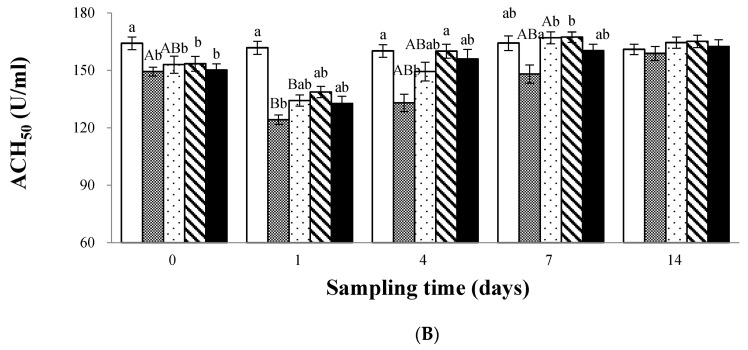
Effects of emodin on white blood counts (**A**) and ACH_50_ (**B**) in *Megalobrama amblycephala* after intraperitoneal injection. Note: Data are expressed as means ± SEM (*n* = 9). Diverse little letters show significant differences (*p* < 0.05) in different dosage groups of each sampling point and diverse capital letters show significant differences (*p* < 0.05) in different time in Duncan’s multiple range test.

Figure 3A shows that the dietary supplementation of emodin significantly affects the WBC of infected Wuchang bream. At the beginning of the experiment, compared with the negative control group, the WBC of the other four groups increased significantly (*p* < 0.05); on day 1, the WBC of the infected groups increased compared to the beginning of the experiment, and the WBC of the 100 mg kg^−1^ emodin group was significantly higher than (*p* < 0.05) the positive control group, while there was no significant difference (*p* > 0.05) in the other groups; on day 4, the WBC of Wuchang bream in the emodin groups decreased compared to the 1st day, but the WBC of the positive control group was still significantly higher than (*p* < 0.05) the negative control group; starting from the 7th day, the WBC of the 100 mg kg^−1^ group decreased to the level of the negative control group and was significantly (*p* < 0.05) lower than the positive control group, with no significant difference in the other groups (*p* > 0.05). Furthermore, the white blood cell count was also significantly affected by the feeding time of emodin, with a similar trend observed as in TNF-α content.

The ACH_50_ activity of the negative control group of the infected Wuchang bream was not affected by the dietary supplementation and feeding time of emodin, and the variation was relatively stable. However, the positive control group and the 30 mg kg^−1^ group were significantly affected by feeding time (*p* < 0.05). The supplementation of 100 and 150 mg emodin kg^−1^ diet could inhibit the decrease in ACH_50_ activity in Wuchang bream after infection (Figure 3B). At the beginning of the experiment, the ACH_50_ activity of the infected groups was significantly lower than that of the negative control group (*p* < 0.05), but there was no significant difference (*p* > 0.05) between the four groups. On the first day, the ACH_50_ activity of the positive control group was significantly lower than that of the negative control group (*p* < 0.05), while there was no significant difference (*p* > 0.05) among the other groups. On the fourth day, the supplementation of 30, 100, and 150 mg kg^−1^ emodin in the diet could increase the ACH_50_ activity of infected Wuchang bream, but the ACH_50_ activity in the positive control group was still significantly lower than that in the negative control group and the 100 mg kg^−1^ group (*p* < 0.05). On the seventh day, there was no significant difference (*p* > 0.05) in ACH_50_ activity between the emodin groups and the negative control group, but the ACH_50_ activity in the positive control group was still significantly lower than that in the 30 and 100 mg kg^−1^ groups (*p* < 0.05). After the 7th day, the ACH_50_ activity of each group except the positive control group remained stable, and by the 14th day, there was no significant difference (*p* > 0.05) in ACH_50_ activity among the groups.

### 3.3. The Antioxidant Status in Plasma

**Figure 4:**
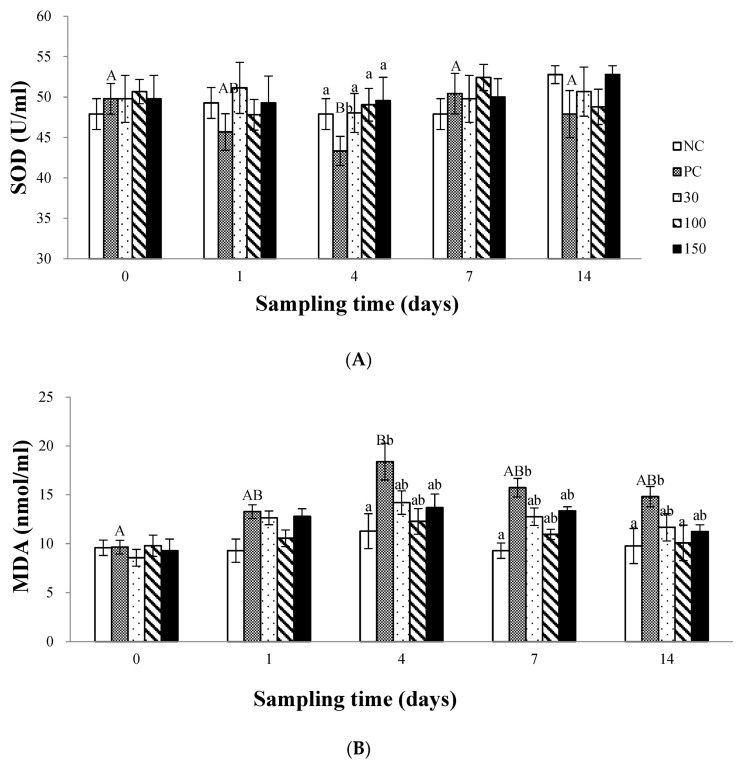
Effects of emodin on Superoxide dismutase (SOD) activity (**A**) and Malonaldehyde (MDA) content (**B**) in *Megalobrama amblycephala* after intraperitoneal injection. Note: Data are expressed as means ± SEM (*n* = 9). Diverse little letters show significant differences (*p* < 0.05) in different dosage groups of each sampling point and diverse capital letters show significant differences (*p* < 0.05) in different time in Duncan’s multiple range test.

During the 14-day experimental period, the SOD activity in the plasma of the infected Wuchang bream was shown in Figure 4A. The results showed no significant differences (*p* > 0.05) in SOD activity in the plasma of the other groups compared to the negative control group at the beginning of the experiment and on days 1, 7, and 14 (*p* > 0.05). On day 4, the SOD activity in the blood plasma of the positive control group was significantly lower than that of the other groups (*p* < 0.05). In addition, the feeding time of the diet did not significantly affect the plasma of all groups except the positive control group (*p* > 0.05).

As shown in Figure 4B, the MDA content in the plasma of the infected Wuchang bream was significantly affected by the dietary supplementation of emodin. At the beginning and on day 1 of the experiment, there was no significant difference (*p* > 0.05) in the MDA content among the groups. On day 4, the plasma MDA content in the emodin groups increased compared to day 1, but there was no significant difference (*p* > 0.05) compared to the negative control group (*p* > 0.05), while the plasma MDA content in the positive control group was significantly lower than that in the negative control group (*p* < 0.05). On day 7, the plasma MDA content in the emodin groups decreased significantly compared to day 4, and there was no significant difference compared to the negative control group, whereas the MDA content in the positive control group remained significantly higher than that in the negative control group (*p* < 0.05). On day 14, the MDA content in the positive control group was significantly higher than that in the negative control group and the 100 mg kg^−1^ group, and there was no significant difference (*p* > 0.05) among the other groups. In addition, the plasma MDA content in the positive control group was significantly affected by the feeding time of the diet (*p* < 0.05), showing an increasing and then decreasing trend, while the MDA content in the other groups was not significantly affected by feeding time.

### 3.4. Histological Examination

**Figure 5:**
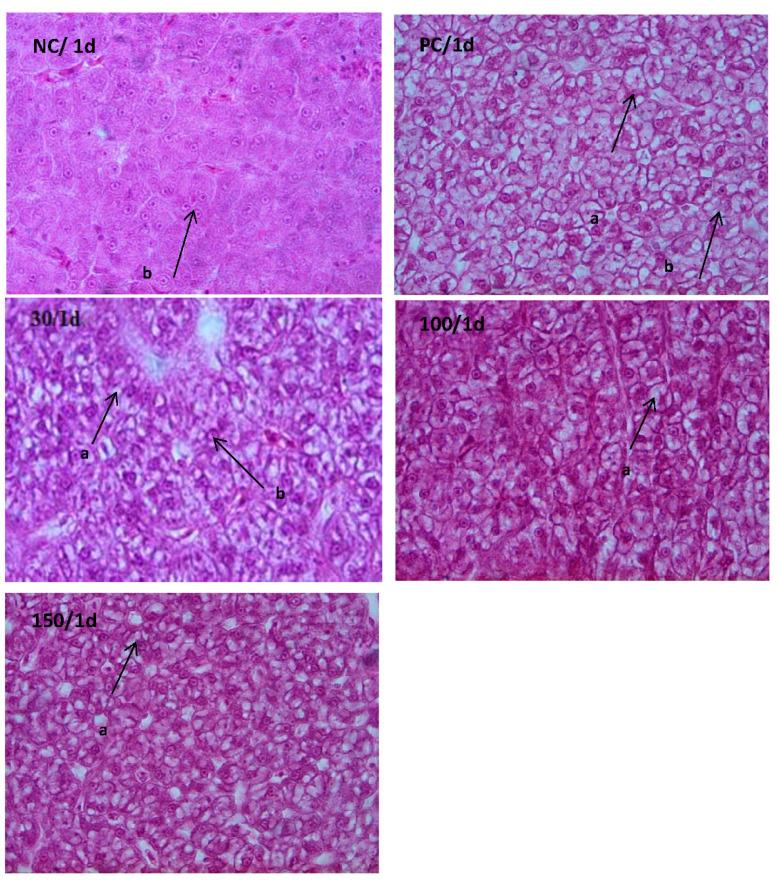
Effects of emodin on liver histology in *Megalobrama amblycephala* on the first day of the experiment (×1000). Note: a indicated adipose hollow space; b indicated cell nucleus.

**Figure 6:**
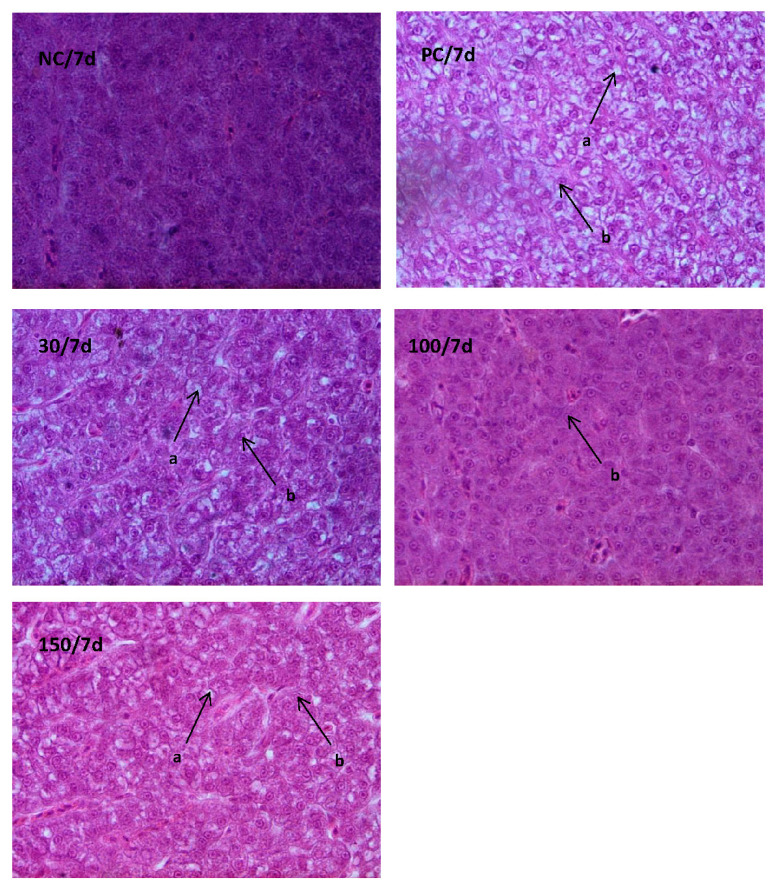
Effects of emodin on liver histology in *Megalobrama amblycephala* on the seventh day of the experiment (×1000). Note: a indicated adipose hollow space; b indicated cell nucleus.

The effects of emodin on the histological structure of the liver in Wuchang bream after intraperitoneal injection of *A. hydrophila* are shown in Figure 5 and Figure 6. On day 1 of the experiment, the liver cells in the infected groups exhibited severe vacuolation and cellular swelling, with most nuclei being displaced. Among them, the positive control group showed the most severe cellular swelling and vacuolation. In contrast, the liver cells in the negative control group had a relatively normal shape, without significant cell swelling or obvious lipid vacuoles. The nucleus size was moderate and distributed in the middle of the cell. On day 7, only the positive control group’s liver cells showed vacuolization and nuclear displacement in some cells. However, compared with day 1, the vacuolation and swelling of the liver cells were reduced. In addition, the shape of the liver cells in the other groups was relatively normal, without significant cell swelling or obvious lipid vacuoles. The nucleus size was moderate and distributed in the middle of the cell.

## 4. Discussion

In this experiment, the total number of *Aeromonas* in the liver, kidney, and blood of infected Wuchang bream showed an upward trend, and the downward trend only appeared after one day of the experiment, which indicates that emodin can kill *A. hydrophila* to some extent and treat diseases caused by *A. hydrophila*, but it is not immediately effective in the early stages of infection. This is consistent with the results of Zheng et al. [24] and Chen et al. [25] in using Chinese herbal medicine to prevent and treat septicemia in *Carassius auratus*. This may be because the digestion and absorption of traditional Chinese medicine in fish is relatively slow, and it needs to enhance the body’s immunity and repair damaged tissues to achieve the effect of treating both symptoms and root causes. Therefore, traditional Chinese medicine takes a longer time to take effect but has a longer duration of action compared to Western medicine. In addition, at the same time point, the quantities of *Aeromonas* in the tissues of the 30, 100, and 150 mg kg^−1^ groups were significantly lower than those in the positive control group. Among them, the 100 mg kg^−1^ group exhibited the lowest count of *Aeromonas*, but the difference compared to the other two groups was not statistically significant. Furthermore, the negative control group showed no significant difference when compared to the 100 mg kg^−1^ group, yet it was significantly lower than the other two groups. This indicates that the addition of emodin to the diet has a bactericidal effect on *A. hydrophila*. However, both excessively low and excessively high concentrations fail to achieve the optimal therapeutic effect. This conclusion is similar to the result of Yang et al. [26] in their study on the treatment of septicemia in grass carp using traditional Chinese herbal medicine. This could be because an insufficient dosage of emodin supplemented to the diet affects the efficacy of the treatment, while an excessive dosage of emodin can result in a strong odor in the feed, significantly impacting the taste and olfactory senses of the fish, leading to decreased feeding rates and diminished therapeutic effects. It is also possible that higher dosages disrupt metabolic functions, upset the digestion and absorption balance, and consequently reduce the fish’s absorption and utilization of emodin [11]. Therefore, it is recommended to supply 100 mg kg^−1^ emodin to the diet of Wuchang bream during the high incidence season of *A. hydrophila* infection to achieve the effect of resisting the infection of *A. hydrophila.*

During the process of phagocytosis of invading foreign substances, phagocytic cells generate a large amount of respiratory burst [27], which can serve as an active indicator of organ clearance of foreign substances. TNF-α is a small molecular protein produced by activated white blood cells, which can promote phagocytosis of granulocytes, inhibit and kill tumor cells, promote cell proliferation and differentiation, and is an important inflammatory factor. Studies by Hardie et al. [28] and Whyte [29] also demonstrate that TNF-α is a macrophage activating factor (MAF), which can stimulate the activity of macrophages and enhance respiratory burst activity. White blood cells are a type of phagocytic cell and an important defense line in the body. When the body undergoes bacterial infections, the number of white blood cells increases significantly, phagocytizing invading bacteria, parasites, and other foreign substances and producing antibodies to cure body damage [30].

In this experiment, the white blood cell count, respiratory burst activity, and TNF-α content in the blood of Wuchang bream infected with *Aeromonas hydrophila* showed an initial increase followed by a decrease. Except for the negative control group, all other groups had the highest white blood cell count, respiratory burst activity, and TNF-α content on the first day of the experiment, indicating that emodin does not immediately produce an effect on infected Wuchang bream. Furthermore, on the first day of the experiment, the white blood cell count, respiratory burst activity, and TNF-α content in the 100 mg kg^−1^ group were significantly higher than those in the positive control group but not significantly higher than those in the 30 and 150 mg kg^−1^ groups. This conclusion is consistent with previous research results [24,29]. This indicates that the supplementation of 30, 100, and 150 mg kg^−1^ emodin to the diet can, to some extent, kill *A. hydrophila*, reduce inflammation, and improve the immune function of the body. However, the pharmacological activity of emodin is complex, and it is speculated that it may play a therapeutic role through the following aspects: (1) Emodin is the main component of the extract of rhubarb anthraquinone, which can cause the strain to produce various toxins, such as hemolysin, enterotoxin, and cytotoxin, directly killing bacteria [31]; (2) Increase the production of white blood cells in the body so that they can engulf a large number of exogenous bacteria; (3) Promote the secretion of TNF-α and induce the production of respiratory burst, activate the body’s defense system, thereby playing a bactericidal role and restoring the body to normal [32]. At the same time, from the fourth day onwards, the white blood cell count, respiratory burst activity, and TNF-α content of the positive control group showed a decreasing trend but still significantly higher than the negative control group, indicating that infected Wuchang bream can achieve a bactericidal effect through their own immune function, but cannot completely eliminate inflammation. However, compared with the positive control group, the 100 mg kg^−1^ group was able to significantly reduce the white blood cell count, respiratory burst activity, and TNF-α content in the shortest time, making them not significantly different from the negative control group. This conclusion demonstrates that supplying 100 mg kg^−1^ emodin to the diet can reduce the body’s inflammation more quickly than supplying 30 and 150 mg kg^−1^, which is beneficial for improving the ability of Wuchang bream to resist *A. hydrophila* infection. This may be because, under the condition of bacterial infection, the body’s own functions are damaged to some extent, and the immune function is reduced. Excessive addition of emodin may increase the burden on the body when the immunity is low, causing damage to the liver and making it difficult for emodin to be well absorbed and utilized. The specific mechanism of action needs further research.

Complement, as a non-specific immune response factor in the body, has the ability to resist bacteria, fungi, viruses, and parasites [33]. Some studies have shown that the extract of rhubarb anthraquinones [34] and emodin [12] can enhance the complement activity of C3 and C4 in Wuchang bream. However, there have been no reports on whether emodin can increase the ACH_50_ activity of Wuchang bream. In this experiment, the ACH_50_ activity of Wuchang bream showed an opposite trend to the white blood cell count and TNF-α content, indicating that emodin activated the bactericidal immune response mechanism in Wuchang bream, such as respiratory burst activity and some humoral or cellular immune factors, thereby initiating the immune defense system of the body and ultimately achieving a normal state.

*A. Hydrophila,* as a bacterial stress factor, can cause great damage to Wuchang bream. Stress can cause the body to produce excessive oxygen free radicals, which, if exceeded in the body’s own tolerance, can disrupt the balance between free radical production and clearance in the body. Excessive free radicals and other reactive oxygen species (ROS) have strong oxidative properties and can attack the double bonds of unsaturated fatty acids in the membrane, increasing the production of lipid peroxides in the body and causing lipid peroxidation damage [35,36]. The antioxidant enzyme system plays a very important role in resisting lipid peroxidation damage [37,38]. In this experiment, supplying 30, 100, and 150 mg kg^−1^ emodin to the diet had no significant effect on the SOD activity and MDA content in the infected Wuchang bream plasma. While the plasma SOD activity in the positive control group was significantly lower than that in the other groups, and its MDA content was significantly higher than that in the other groups. Studies have shown that supplying Chinese herbal extracts to aquatic animal diets may increase antioxidant capacity to resist the effects of stress [5,39]. This is opposite to our conclusion, which may be due to the fact that when the organism is infected with pathogenic bacteria, the ROS induced by emodin is used to fully kill external bacteria and microorganisms, maintain the dynamic balance of the organism’s oxidation and antioxidant system, and protect the organism from lipid peroxidation damage so it does not cause changes in the relevant antioxidant enzyme system.

The liver is the most important metabolic organ and the largest digestive gland in fish, which can participate in various functions, including digestion, metabolism, excretion, detoxification, and immunity. In this experiment, during the early stages of *A. hydrophila* infection in Wuchang bream, liver cells showed vacuolization and nuclear displacement. However, after feeding with a diet containing emodin for a period of time, liver cells in the 100 mg kg^−1^ group no longer showed vacuolization, while those in the 30 mg kg^−1^ and 150 mg kg^−1^ groups showed slight vacuolization. This indicates that emodin has a certain reparative effect on liver damage in Wuchang fish, with the best effect observed at a dosage of 100 mg kg^−1^. Similar conclusions have been reported in humans [40] and mice [35]. This may be because emodin can induce the production of a large amount of ROS in the body during the early stages of infection, which can cause damage to damaged cell DNA and achieve the goal of repairing liver damage by regulating the expression of apoptosis-related genes [41]. However, insufficient or excessive use of emodin cannot achieve optimal results.

In conclusion, the supplementation of 100 mg kg^−1^ of emodin in the diet can enhance the phagocytic function of the body, promote the secretion of cytokines, and kill pathogenic bacteria in the body, thereby improving the resistance of Wuchang bream to *A. hydrophila* infection and restoring the immune function of the body. Moreover, compared to other doses, 100 mg kg^−1^ emodin has the best repair effect on liver damage caused by exogenous bacteria in Wuchang bream. This study has preliminarily explored the repair mechanism of emodin on Wuchang bream infection, providing theoretical guidance for the further development of green hepatoprotective drugs for fish.

## Figures and Tables

**Table 1:** Formulation and proximate composition of the basal diet (%).

Ingredients	Percentage Dry Weight
Fish meal	8
Soybean meal	18
Rapeseed meal	17
Cotton meal	16.5
Rice bran	8
Wheat middling	22
Soybean oil	4
Lecithin	1
Choline chloride	0.5
Vitamin premix ^a^	1
Mineral premix ^b^	1
Powdered zeolite	1
Calcium dihydrogen phosphate	2
Proximate composition (%)	
Crude protein	31.27
Crude lipid	8.15
Crude ash	11.02
Gross energy (kJ g^−1^) ^c^	16.32

^a^ Vitamin premix (IU or mg per kg premix): Vitamin A, 900,000 IU; Vitamin D, 250,000 IU; Vitamin E, 100 mg; Vitamin K_3_, 220 mg; Vitamin C, 5000 mg; Vitamin B_1_, 320 mg; Vitamin B_2_, 1090 mg; Vitamin B_6_, 5000 mg; Vitamin B_12_, 116 mg; biotin, 50 mg; Pantothenate, 1000 mg; Folic acid, 165 mg; Choline, 60,000 mg; Inositol, 15,000 mg; Niacin acid, 2500 mg. ^b^ Mineral premix (per kg premix): blue copperas, 2.5 g; green vitriol, 28 g; zinc sulfate heptahydrate, 22 g; Manganese sulfate tetrahydrate, 9 g; sodium selenate, 0.045 g; potassium iodide, 0.026 g; cobalt chloride hexahydrate, 0.1g. ^c^ Energy, calculated by using standard physiological fuel values of 17.15, 23.64, and 39.54 kJ/g for carbohydrate, protein, and lipid, respectively.

**Table 2:** Components of the RS selective media.

RS Medium *	(g/L)
L-Ornithine hydrochloride	6.5
L-Lysine hydrochloride	5.0
L-Cysteine hydrochloride	0.3
Maltose	3.5
Sodium thiosulfate	6.8
Ferric ammonium citrate	0.8
Sodium chloride	5.0
Sodium deoxycholate	1.0
Yeast extract	3.0
Bromothymol blue	0.03
novobiocin	0.005

Notes: * Medium contained agar (12.5 g/L), and its ingredients were dissolved in 1000 mL distilled water. The pH of Rimler-Shotts Medium was 7.0 ± 0.1. Autoclave at 120 °C in 20 min.

## Data Availability

All data generated or used during the study appear in the submitted article.
